# Hemodynamic impact of ephedrine on hypotension during general anesthesia: a prospective cohort study on middle-aged and older patients

**DOI:** 10.1186/s12871-023-02244-4

**Published:** 2023-08-22

**Authors:** Yuta Uemura, Michiko Kinoshita, Yoko Sakai, Katsuya Tanaka

**Affiliations:** 1 Department of Anesthesiology, Tokushima University Graduate School of Biomedical Sciences, 3-8-15 Kuramoto-cho, Tokushima-shi, Tokushima, 770-8503 Japan; 2 Department of Anesthesiology, Tokushima University Hospital, 2-50-1 Kuramoto-cho, Tokushima-shi, Tokushima, 770-8503 Japan; 3 Division of Anesthesiology, Tokushima University Hospital, 2-50-1 Kuramoto-cho, Tokushima-shi, Tokushima, 770-8503 Japan

**Keywords:** Aging, Elderly, Ephedrine, Hemodynamics, Hypotension

## Abstract

**Background:**

Ephedrine is a mixed α- and β-agonist vasopressor that is frequently used for the correction of hypotension during general anesthesia. β-responsiveness has been shown to decrease with age; therefore, this study aimed to determine whether aging would reduce the pressor effect of ephedrine on hypotension during general anesthesia.

**Methods:**

Seventy-five patients aged ≥ 45 years were included in this study, with 25 patients allocated to each of the three age groups: 45–64 years, 65–74 years, and ≥ 75 years. All patients received propofol, remifentanil, and rocuronium for the induction of general anesthesia, followed by desflurane and remifentanil. Cardiac output (CO) was estimated using esCCO technology. Ephedrine (0.1 mg/kg) was administered for the correction of hypotension. The primary and secondary outcome measures were changes in the mean arterial pressure (MAP) and CO, respectively, at 5 min after the administration of ephedrine.

**Results:**

The administration of ephedrine significantly increased MAP (*p* < 0.001, mean difference: 8.34 [95% confidence interval (CI), 5.95–10.75] mmHg) and CO (*p* < 0.001, mean difference: 7.43 [95% CI, 5.20–9.65] %) across all groups. However, analysis of variance revealed that the degree of elevation of MAP (F [2, 72] = 0.546, *p* = 0.581, η^2^ = 0.015 [95% CI, 0.000–0.089]) and CO (F [2, 72] = 2.023, *p* = 0.140, η^2^ = 0.053 [95% CI, 0.000–0.162]) did not differ significantly among the groups. Similarly, Spearman’s rank correlation and multiple regression analysis revealed no significant relation between age and the changes in MAP or CO after the administration of ephedrine.

**Conclusion:**

The administration of ephedrine significantly increased MAP and CO; however, no significant correlation with age was observed in patients aged > 45 years. These findings suggest that ephedrine is effective for the correction of hypotension during general anesthesia, even in elderly patients.

**Trial registration:**

UMIN-CTR (UMIN000045038; 02/08/2021).

**Supplementary Information:**

The online version contains supplementary material available at 10.1186/s12871-023-02244-4.

## Background

Hypotension is common during general anesthesia and is associated with postoperative mortality and morbidity [1–3]. Even a short period of intraoperative hypotension is associated with acute kidney injury and cardiovascular events [4, 5]. Vulnerability to hemodynamic disturbances increases with age; therefore, fast-acting treatments must be administered to elderly patients who develop hypotension during anesthesia [6, 7].

Ephedrine, a mixed α-adrenergic and β-adrenergic receptor agonist, is frequently used for the correction of hypotension during general anesthesia [8, 9]. Previous studies have reported that ephedrine maintains or increases the cardiac output (CO) by stimulating the β-adrenergic receptor, which may improve tissue perfusion and oxygenation [10–12]. Thus, ephedrine is expected to have a more favorable profile than pure α-agonists, such as phenylephrine, for the treatment of hypotension [13]. However, elderly patients may derive fewer benefits from ephedrine [14] as preclinical studies have indicated that potential factors related to advanced age may result in a reduced hemodynamic response to β-agonists [15–17]. No clinical studies have examined the age-related differences in the hemodynamic effect of ephedrine.

Therefore, this study aimed to determine whether aging would reduce the pressor effects of ephedrine on hypotension during general anesthesia. The primary objective of this study was to examine the age-related differences in the increase in the mean arterial pressure (MAP) after the administration of ephedrine. The secondary objective was to evaluate the age-related differences in the increase in CO after the administration of ephedrine. In addition, the changes in systolic blood pressure (SBP), diastolic blood pressure (DBP), heart rate (HR), and stroke volume (SV) were also evaluated as secondary outcomes.

## Methods

### Study design and ethics

This prospective observational cohort study was approved by the Tokushima University Hospital Ethics Committee (approval number: 4000; 28/06/2021) and registered with the University Hospital Medical Information Network Clinical Trial Registry (UMIN000045038; 02/08/2021). All patients provided written informed consent prior to the start of the study. This work adheres to the Strengthening the Reporting of Observational Studies in Epidemiology (STROBE) statement. The administration of ephedrine was justified only for patients who presented with hypotension.

### Patient recruitment

This single-center study was conducted at the Tokushima University Hospital between July and November 2021. Seventy-five patients aged ≥ 45 years were included in this study, with 25 patients allocated to each of the following three age groups: middle age group, 45–64 years of age; early elderly group, 65–74 years of age; and late elderly group, ≥ 75 years of age. Only patients aged ≥ 45 years were included in this study as distinct patient profiles were anticipated between the younger and middle-aged to older age groups. Consecutive patients who received ephedrine during the observation period starting from the earliest date of surgery were included until the sample size reached 25 patients in each group.

The eligibility criteria were as follows: ≥45 years of age; American Society of Anesthesiologists Physical Status I or II; and scheduled to undergo surgery under general anesthesia. All potentially eligible patients were evaluated in advance at the outpatient anesthesiology department. The exclusion criteria were as follows: surgery performed in a position other than the supine position; the presence of cardiovascular, respiratory, or severe mental disease; the presence of neurological disorder or severe liver dysfunction; electroconvulsive therapy; dialysis; pregnancy; obesity (body mass index [BMI] ≥ 35 kg/m^2^) or emaciation (BMI < 14 kg/m^2^); and estimated continuous CO (esCCO; Nihon Kohden, Tokyo, Japan) that was deemed difficult to measure. Patients who did not receive ephedrine during the observation period were also excluded.

### General anesthesia, data collection, and outcomes

**Fig. 1:**
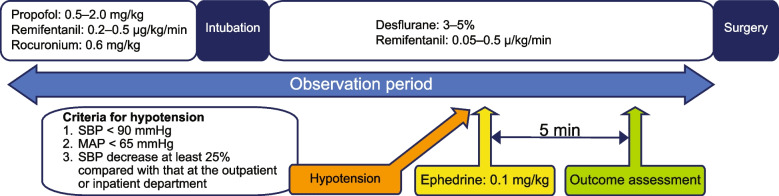
Study protocol. SBP, systolic blood pressure; MAP, mean arterial pressure

Patients, except for those scheduled to undergo gastrointestinal surgery, received oral rehydration solution (Arginaid Water®; Nestle Japan, Kobe, Japan) as needed until 2 to 3 h before entering the operating room. Propofol (0.5–2.0 mg/kg), remifentanil (0.2–0.5 µg/kg/min), and rocuronium (0.6 mg/kg) were administered for the induction of general anesthesia, followed by desflurane (3–5%) and remifentanil (0.05–0.5 µ/kg/min) to maintain the bispectral index value at 40–60. Endotracheal intubation or supraglottic instrument insertion was performed, followed by mechanical ventilation to maintain the end-tidal CO_2_ levels at 35–45 mmHg. Ringer’s solution acetate (10 mL/kg/h) was administered through a secured intravenous line. The pulse wave transit time was measured to estimate CO and SV using esCCO technology. The blood pressure was measured non-invasively with an upper arm cuff every 2.5 min. Ephedrine (0.1 mg/kg) was administered to patients who presented with hypotension. Patients who satisfied any of the following conditions were considered to have hypotension: SBP < 90 mmHg, MAP < 65 mmHg, or a decrease in SBP of at least 25% compared with that measured in the outpatient or inpatient department. The blood pressure measured in the outpatient department referred to measurements obtained by the patients themselves at any hour during the daytime using an automatic sphygmomanometer, whereas blood pressure measured in the inpatient department referred to the measurements obtained by nurses on the morning of the day of the surgery. If the patient met the hypotension criteria 5 min after the administration of ephedrine, phenylephrine (0.002 mg/kg) was administered to correct hypotension. Further treatment was provided at the discretion of the anesthesiologist in charge if no increase in the blood pressure was observed after the administration of phenylephrine. The observation period ranged from anesthesia induction until the start of surgery. Figure 1 presents an overview of the study protocol.

The primary outcome measure was the age-related differences in the change in MAP 5 min after the administration of ephedrine. The secondary outcomes were the age-related differences in the change in CO 5 min after the administration of ephedrine and the changes in SBP, DBP, HR, and SV.

### Sample size

The sample size was calculated by assuming a MAP of 80 mmHg (standard deviation [SD], 10 mmHg) after the administration of ephedrine, as described previously [8, 10]. A 10% difference in hemodynamics was considered a significant clinical change [10, 12]. Therefore, we aimed to detect a clinically important difference of 0.8 SD in the primary outcome measure. The effect size (d) of 0.8 was converted to an effect size (f) of 0.4 using the esc package in R version 4.1.3 (The R Foundation for Statistical Computing, Vienna, Austria). Using an effect size (f) of 0.4, α error of 0.05, and power of 0.85, the sample size was calculated as 72 using G*Power version 3.1.9.6 (Heinrich-Heine-Universität Düsseldorf, Germany) [18]. However, the sample size was rounded to 75 patients (*n* = 25 per age group).

### Statistical analysis

Data are presented as the mean (SD) or number (%). Numerical variables were assessed using a one-way or repeated-measures analysis of variance (ANOVA). Binary variables were assessed using the chi-square test, and Fisher’s exact test was performed when there were five or fewer cells. The F-statistic and the effect size (η²) were reported for ANOVA, along with the 95% confidence interval (CI), for both primary and secondary outcomes. Two sensitivity analyses were performed subsequently. The association between age and the changes in MAP and CO was examined using Spearman’s rank correlation coefficient. The following four factors that could potentially affect changes in MAP and CO were examined using a multiple regression analysis: age; BMI related to ephedrine dosage; medication for hypertension with angiotensin-converting enzyme inhibitors (ACEIs)/angiotensin receptor blockers (ARBs); and the administration of preoperative oral rehydration solution. Variance inflation factors were calculated for each variable to address the potential issue of multicollinearity, which can pose problems in multiple regression analysis; none of the factors were found to exceed 5. Statistical significance was set at *p* < 0.05 (two-sided). Statistical analyses were performed using R version 4.1.3 (The R Foundation for Statistical Computing) with EZR (Saitama Medical Center, Jichi Medical University, Saitama, Japan) [19].

## Results

**Fig. 2:**
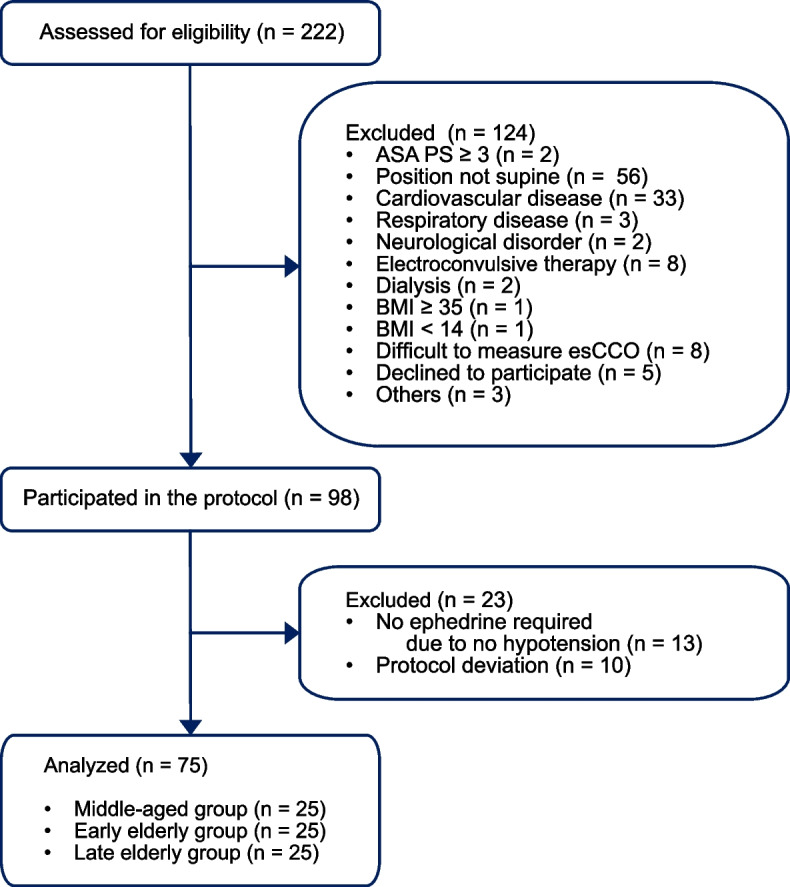
Flow diagram. n, number; ASA PS, American Society of Anesthesiologist Physical Status; BMI, body mass index; esCCO, estimated continuous cardiac output

Among the 222 patients initially assessed for eligibility, 124 patients were excluded after the application of the aforementioned exclusion criteria or due to refusal to participate in the study. Among the remaining 98 patients, 23 patients were excluded as ephedrine was not administered to these patients, they had insufficient available data, or their treatment deviated from the protocol. The remaining 75 patients were allocated to one of the three age groups (middle age, early elderly, and late elderly groups) (Fig. 2). Table 1 presents the patient characteristics. The youngest patient was 46 years old, whereas the oldest patient was 87 years old. Older patients tended to have a higher American Society of Anesthesiologist Physical Status and required a lower dosage of propofol and remifentanil for the induction of anesthesia.

**Table 1:** Patients’ characteristics

	Middle age (45–64 years) *n* = 25	Early elderly (65–74 years) *n* = 25	Late elderly (≥ 75 years) *n* = 25	*p* values
Age, years	55.6 (5.7)	70.3 (2.6)	79.4 (3.5)	
Sex, male/female	7/18	12/13	11/14	0.311
BMI, kg/m^2^	23.4 (3.6)	22.8 (4.0)	24.0 (2.8)	0.518
ASA PS, I/II	8/17	4/21	1/24	0.036
Use of ACEIs/ARBs	1	4	5	0.319
Anesthetics for induction				
Propofol, mg/kg	1.4 (0.2)	1.3 (0.2)	1.1 (0.2)	< 0.001
Remifentanil, µg/kg/min	0.32 (0.06)	0.29 (0.04)	0.28 (0.04)	0.016
Anesthetics for maintenance				
Desflurane, %	4.0 (0.5)	3.9 (0.5)	3.7 (0.3)	0.078
Remifentanil, µg/kg/min	0.23 (0.05)	0.23 (0.07)	0.20 (0.05)	0.135
Surgery type				
Gastrointestinal surgery	3	1	10	
Orthopedic surgery	2	1	0	
ENT surgery	5	8	1	
Ophthalmology surgery	1	2	4	
Urologic surgery	2	3	6	
Breast surgery	6	6	2	
Gynecological surgery	6	4	2	

Data are expressed as mean (standard deviation) or number

*ACEIs *Angiotensin-converting enzyme inhibitors, *ARBs *Angiotensin receptor blockers, *ASA PS *American Society of Anesthesiologists Physical Status, *BMI *Body mass index, *ENT *Ear, nose, throat; *n *number

Fourteen, 12, and 12 patients from the middle-aged, early elderly, and late elderly groups, respectively, were found to satisfy the hypotension criterion of SBP < 90 mmHg. Most patients who satisfied the SBP-based hypotension criterion also satisfied the MAP-based hypotension criterion. Thus, including duplicates, eight, 10, and eight patients from the middle-aged, early elderly, and late elderly groups, respectively, were considered to have satisfied the hypotension criterion of MAP < 65 mmHg. In addition, 11, 12, and 12 patients from the middle-aged, early elderly, and late elderly groups, respectively, satisfied the hypotension criterion of showing a decrease in SBP of at least 25% compared with the measurement taken in the outpatient or inpatient department.

Table 2 presents the hemodynamic changes in MAP and CO during the observation period. No significant differences in MAP were observed among the age groups at baseline (F [2, 72] = 0.259; *p* = 0.773), the time of hypotension (F [2, 72]= 0.818; *p* = 0.446), and 5 min after the administration of ephedrine (F [2, 72] = 0.128; *p* = 0.880). Similarly, no significant differences in CO were observed among the age groups at the time of hypotension (F [2, 72] = 0.152; *p* = 0.860) and 5 min after the administration of ephedrine (F [2, 72] = 0.735; *p* = 0.483). The administration of ephedrine significantly increased MAP (*p* < 0.001; mean difference, 8.34 mmHg; 95% CI, 5.95–10.75 mmHg) and CO (*p* < 0.001; mean difference, 7.43%; 95% CI, 5.20–9.65%) in all groups. However, the increases in MAP (F [2, 72] = 0.546; *p* = 0.581; η^2^ = 0.015; 95% CI, 0.000–0.089) and CO (F [2, 72]  = 2.023; *p* = 0.140; η^2^ = 0.053; 95% CI, 0.000–0.162) did not differ significantly among the age groups.

**Table 2:** Hemodynamic changes during the observation period among the patient groups

	Middle age (45–64 years) *n* = 25	Early elderly (65–74 years) *n* = 25	Late elderly (≥ 75 years) *n* = 25	*p* values
MAP, mmHg				
Before anesthesia induction (baseline)	107.4 (14.5)	108.6 (9.5)	106.1 (12.6)	0.773
At the time of hypotension	70.6 (10.6)	67.3 (9.5)	68.5 (7.2)	0.446
At 5 min after ephedrine administration	78.0 (13.5)	77.5 (15.0)	76.1 (12.3)	0.880
Change after ephedrine administration	7.3 (9.4)	10.1 (11.0)	7.6 (11.0)	0.581
CO (%)				
Before anesthesia induction (baseline)	100	100	100	1
At the time of hypotension	74.5 (16.0)	76.3 (15.2)	74.1 (13.8)	0.860
At 5 min after ephedrine administration	79.5 (15.6)	83.3 (15.6)	84.4 (14.2)	0.483
Change after ephedrine administration	5.0 (9.2)	7.0 (11.1)	10.3 (8.0)	0.140

Data are expressed as mean (standard deviation)

*CO *Cardiac output, *MAP *Mean arterial pressure, *n *number

Supplemental Table 1 presents the hemodynamic changes in SBP, DBP, HR, and SV. No significant differences were observed in SBP, DBP, HR, and SV among the age groups at baseline, the time of hypotension, and 5 min after the administration of ephedrine. The administration of ephedrine significantly increased SBP (*p* < 0.001; mean difference, 12.91 mmHg; 95% CI, 9.49–16.32 mmHg), DBP (*p* < 0.001; mean difference, 6.07 mmHg; 95% CI, 3.93–8.21 mmHg), HR (*p* = 0.002; mean difference, 3.79 bpm; 95% CI, 1.49–6.09 bpm), and SV (*p* < 0.001; mean difference, 3.49%; 95% CI, 2.48–4.50%) in all groups. However, the increases in SBP, DBP, HR, and SV did not differ significantly among the age groups (*p* = 0.884, *p* = 0.348, *p* = 0.249, *p* = 0.808, respectively).

**Fig. 3:**
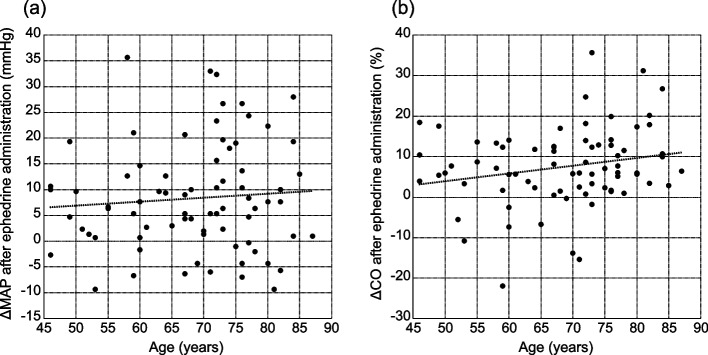
Spearman’s rank correlation coefficients for age and the changes in MAP (**a**) and CO (**b**). Correlations between age and the changes in MAP (correlation coefficient, 0.056; *p* = 0.633) and CO (correlation coefficient, 0.191; *p* = 0.100). Although the correlation coefficient and *p* value were calculated using Spearman’s rank correlation, the linear regression line is presented in this graph. ΔMAP, change in the mean arterial pressure; ΔCO, change in the cardiac output. MAP, mean arterial pressure; CO, cardiac output

Spearman’s rank correlation coefficients were not statistically significant between age groups and the changes in MAP (*r* = 0.056; *p* = 0.633) and CO (*r* = 0.191; *p* = 0.100) (Fig. 3). Multiple regression analysis revealed no significant relationship between age and the changes in MAP (*p* = 0.680) and CO (*p* = 0.186). BMI was significantly associated with changes in CO following the administration of ephedrine (*p* = 0.025). The administration of ACEIs/ARBs and preoperative rehydration solutions had no significant impact on the hemodynamic changes following the administration of ephedrine administration (Table 3).

**Table 3:** Multiple regression analysis of MAP and CO changes after ephedrine administration

Variables	Regression coefficient	95% CI	*p* value	VIF
MAP change, mmHg				
Age, years	0.050	−0.184 to 0.282	0.680	1.11
BMI, kg/m^2^	0.653	−0.032 to 1.339	0.062	1.06
ACEIs/ARBs, binary	5.035	−2.085 to 12.156	0.163	1.03
Preoperative oral rehydration solution intake, binary	−1.126	−7.382 to 5.130	0.721	1.07
CO change, %				
Age, years	0.142	−0.070 to 0.353	0.186	1.11
BMI, kg/m^2^	0.716	0.094 to 1.339	0.025	1.06
ACEIs/ARBs, binary	0.361	−6.098 to 6.819	0.912	1.03
Preoperative oral rehydration solution intake, binary	3.900	−1.775 to 9.575	0.175	1.07

*ACEIs *Angiotensin converting enzyme inhibitors, *ARBs *Angiotensin receptor blockers, *BMI *Body mass index, *CI *Confidence interval, *CO *Cardiac output, *MAP *Mean arterial pressure, *VIF *Variance inflation factor

Phenylephrine was administered to 10, 10, and seven patients from the middle-aged, early elderly, and late elderly groups, respectively, as they satisfied the hypotensive criterion used in this study despite the administration of ephedrine.

## Discussion

We examined the hemodynamic responses after the administration of ephedrine for the correction of hypotension during general anesthesia among different age groups. The administration of ephedrine significantly increased MAP and CO; however, there were no significant age-related differences in these increases among the different age groups. Similarly, no significant differences were observed in SBP, DBP, HR, or SV among the different age groups. The effect sizes (η^2^) for the changes in MAP and CO among the groups were 0.015 and 0.053, respectively, and equivalent to the effect sizes (f) of 0.123 and 0.237, respectively [20, 21]; however, they were considerably smaller than the target of 0.4, which indicated a clinically important difference, set in this study. Moreover, the sensitivity analyses confirmed that there were no significant relationships between age and the changes in MAP and CO after the administration of ephedrine. BMI was related to the changes in CO after the administration of ephedrine, possibly because the ephedrine dose was determined based on body weight. Previous studies have shown the dose-dependent effect of ephedrine [22, 23]. Preoperative administration of oral rehydration solution showed no significant impact on the pressor effect of ephedrine. This finding is consistent with those of previous reports asserting that preoperative fluid optimization via the administration of oral rehydration solution or infusion loading did not contribute to hemodynamic stability following the induction of anesthesia [24, 25].

Aging is known to impair exercise-induced and pharmacological stimuli-induced increases in CO [26, 27]. Previous studies have shown that β-adrenergic receptors become desensitized with age; this effect was mediated by the reduced density of β-adrenergic receptors, reduced G-proteins, and impaired β-adrenergic receptor, G-protein, and adenylyl cyclase activity [27]. Although previous studies have indicated that aging may impair the physical response to β-adrenergic stimuli, the results of the present study suggest that the pressor effect of ephedrine during anesthesia does not vary significantly with age, or at least not to a degree that is clinically significant. Our findings are consistent with those of previous studies that demonstrated that prophylactic ephedrine attenuated circulation suppression after the induction of anesthesia or subarachnoid anesthesia, even in elderly patients [22, 28–30]. To our knowledge, this is the first study to examine the pressor effect of ephedrine according to age.

This study could not elucidate the reason for the absence of age-related differences in the pressor effect of ephedrine despite the decrease in β-responsiveness with age. This finding may be attributed to the following reasons. This study examined patients undergoing surgeries under general anesthesia, and the effects of ephedrine observed in anesthetized patients may be greater than those observed in non-anesthetized patients [31, 32]. Propofol and enflurane have been reported to augment pressor responses to ephedrine [31–33], and we used propofol for anesthesia induction in this study.

The main contributor of hypotension during anesthesia induction has been reported to be the decreases in CO and systemic vascular resistance among elderly and young patients, respectively [34]. Furthermore, ephedrine, but not phenylephrine, improves tissue perfusion and oxygenation by maintaining or increasing CO [10–12]. Thus, our finding that ephedrine may increase CO regardless of age has clinically important implications.

Ephedrine acts directly and indirectly on adrenergic receptors [35, 36]. Its in vivo pressor effects depend predominantly on indirect actions, specifically, the release of norepinephrine from the sympathetic nerves [37]. Thus, the pressor effects of indirect adrenergic agonists such as ephedrine depend on the quantity of norepinephrine released from the sympathetic nerves, which is altered by patient-specific circumstances and medications [38].

This study had some limitations. First, the outcomes were measured 5 min after the administration of ephedrine. A longer observation period would have resulted in a more detailed study. However, previous studies have reported that even brief hypotension persisting for only 5 min is significantly associated with postoperative complications [4]. In addition, circulatory changes after the administration of ephedrine are maximal at 2 to 3 min and persist for 5 min [11]. Therefore, the outcomes of this study were measured at 5 min after the administration of ephedrine. Second, non-invasive blood pressure measurements were obtained at intervals of 2.5 min. The continuous measurement of invasive arterial pressure would have provided more detailed data; however, this was deemed inappropriate because of the patients’ characteristics. Third, only patients aged > 45 years were included in this study. Younger patients were not examined as they are at lower risk of developing hypotension during anesthesia and postoperative complications. Therefore, this study included middle-aged and elderly patients who were deemed to require ephedrine. Fourth, CO was estimated using the esCCO system. The accuracy of esCCO is clinically acceptable and comparable with that of both thermodilution CO measurements and arterial pulse contour-based CO measurements [39–41]. As esCCO appears to be better for assessing relative values than absolute values, CO was assessed using percent changes [40, 41].

## Conclusions

In conclusion, increases in MAP and CO caused by the administration of ephedrine were not significantly related to age among patients older than 45 years of age. These results suggest that ephedrine effectively corrects hypotension during general anesthesia, even in elderly patients.

### Supplementary Information

**Additional file 1: Supplemental Table 1.** Hemodynamic changes in the patient groups during the observation period. 

## Data Availability

The datasets used and/or analyzed during the study are available from the corresponding author on reasonable request.

## Abbreviations

ACEIs: Angiotensin converting enzyme inhibitors
ARBs: Angiotensin receptor blockers
BMI: Body mass index
CO: Cardiac output
DBP: Diastolic blood pressure
esCCO: Estimated continuous cardiac output
HR: Heart rate
MAP: Mean arterial pressure
SBP: Systolic blood pressure
SV: Stroke volume
