# Supplementary material for: Hemodynamic impact of ephedrine on hypotension during general anesthesia: a prospective cohort study on middle-aged and older patients
Source: BMC Anesthesiol. 2023 Aug 22;23:283. doi: 10.1186/s12871-023-02244-4 (PMC10464275; doi:10.1186/s12871-023-02244-4)
Supplement: Supplementary file 1 — Additional file 1: Supplemental Table 1. Hemodynamic changes in the patient groups during the observation period. [file 12871_2023_2244_MOESM1_ESM.docx]

Supplemental Table 1. Hemodynamic changes in the patient groups during the observation period

|  | Middle age (45–64 years) n = 25 | Early elderly (65–74 years) n = 25 | Late elderly (≥75 years) n = 25 | p values |
| --- | --- | --- | --- | --- |
| SBP, mmHg |  |  |  |  |
| Before anesthesia induction (baseline) | 145.1 (22.3) | 149.6 (17.0) | 151.6 (20.6) | 0.507 |
| At the time of hypotension | 90.9 (11.4) | 89.4 (12.4) | 90.2 (10.3) | 0.889 |
| At 5 min after ephedrine administration | 102.7 (18.0) | 103.3 (19.7) | 103.4 (18.1) | 0.991 |
| Change after ephedrine administration | 11.8 (14.0) | 13.8 (14.9) | 13.1 (16.1) | 0.884 |
|  |  |  |  |  |
| DBP, mmHg |  |  |  |  |
| Before anesthesia induction (baseline) | 88.6 (11.9) | 88.1 (8.5) | 83.4 (9.7) | 0.139 |
| At the time of hypotension | 60.5 (10.7) | 56.3 (8.9) | 57.6 (7.0) | 0.256 |
| At 5 min after ephedrine administration | 65.6 (11.7) | 64.6 (13.9) | 62.4 (10.2) | 0.638 |
| Change after ephedrine administration | 5.1 (8.2) | 8.3 (10.1) | 4.8 (9.5) | 0.348 |
|  |  |  |  |  |
| HR, bpm |  |  |  |  |
| Before anesthesia induction (baseline) | 77.8 (13.9) | 72.2 (12.2) | 69.5 (11.0) | 0.061 |
| At the time of hypotension | 63.6 (12.7) | 59.4 (11.0) | 57.7 (11.8) | 0.204 |
| At 5 min after ephedrine administration | 66.0 (12.7) | 61.8 (10.3) | 64.2 (14.4) | 0.499 |
| Change after ephedrine administration | 2.4 (6.0) | 2.4 (10.7) | 6.5 (12.1) | 0.249 |
|  |  |  |  |  |
| SV (%) |  |  |  |  |
| Before anesthesia induction (baseline) | 100 | 100 | 100 | 1 |
| At the time of hypotension | 90.1 (4.0) | 90.7 (8.2) | 89.6 (8.9) | 0.855 |
| At 5 min after ephedrine administration | 93.3 (4.7) | 94.7 (8.4) | 92.9 (9.3) | 0.697 |
| Change after ephedrine administration | 3.2 (4.6) | 4.0 (5.0) | 3.4 (3.6) | 0.808 |

Data are expressed as mean (standard deviation).

SBP, systolic blood pressure; DBP, diastolic blood pressure; HR, heart rate; SV, stroke volume; n, number.
